# The Diagnostic Approach to Central Adrenocortical Insufficiency (CAI) in Thalassemia

**DOI:** 10.4084/MJHID.2016.026

**Published:** 2016-05-01

**Authors:** Vincenzo De Sanctis, Heba Elsedfy, Ashraf T Soliman, Ihab Zaki Elhakim, Nada A. Soliman, Mehran Karimi, Rania Elalaily

**Affiliations:** 1 Pediatric and Adolescent Outpatient Clinic, Quisisana Hospital, Ferrara, Italy; 2 Department of Pediatrics, Ain Shams University, Cairo, Egypt; 3 Department of Pediatrics, Division of Endocrinology, Alexandria University Children’s Hospital, Alexandria; 4 Ministry of Health, Alexandria, Egypt; 5 Hematology Research Center, Shiraz University of Medical Science, Shiraz, Iran; 6 Department of Primary Health Care, Abu Nakhla Hospital, Doha, Qatar

In thalassemia major (TM) patients, impairment of the hypothalamic-pituitary-adrenal (HPA) axis secondary to hemosiderosis of the pituitary gland and/or adrenal glands is well established. In TM, hypocortisolism is paucisymptomatic or causes nonspecific symptoms. Although adrenal insufficiency (AI) is rare; an acute crisis may occur in the event of acute cardiac decompensation, stress, or sepsis.^1^–^3^ Furthermore, screening for adrenal insufficiency is commonly overlooked by physicians who manage patients with thalassemia major. The Authors report their experience in the diagnostic utility of glucagon stimulation test (GST) for the diagnosis of central adrenal insufficiency (CAI) and debate the cut-off cortisol level commonly used for the diagnosis of CAI.

The pathophysiological basis of AI in TM has not been well-defined, and there are currently no clear guidelines on how to diagnose AI in these patients.^4^

The diagnosis of CAI is relatively simple when glucocorticoid secretion is profoundly depressed. However, basal cortisol level may be normal in partial CAI and stimulation tests are then necessary to investigate the integrity of the HPA axis and establish the diagnosis.^5^

The standard tests for diagnosing CAI are the insulin hypoglycemia test (ITT) and the metyrapone test (MT). ITT remains the gold standard procedure for the diagnosis of HPA insufficiency. However, it requires close surveillance because of inherent risks of severe hypoglycemia. It also has specific contraindications in patients with epilepsy and heart disease. Therefore, ITT requires close supervision and appears to be demanding for some patients and medical staff.^5^ Moreover, for regularly accepted cut-off points, false positive results are documented, even in normal volunteers, and reproducibility is far from perfect.^6^ A cortisol response < 18 μg/dL (< 500 nmol/l ) has been defined as an evidence of deficiency.^5^

When ITT is contraindicated and where the compound is available the MT test may be performed. Metyrapone inhibits 11β-hydroxylase and, hence, the conversion of 11-DOC into cortisol. Thereby it reduces the negative feedback and triggers ACTH release which, in turn, increases 11-DOC production. Although TM is an excellent test, its use is limited by the difficulty involved in obtaining the medication in many countries and the risk of precipitating an adrenal crisis.^5^ The sum of cortisol and DOC after MT should exceed 16.5 μg/dl (455 nmol/l).^5^

Recent reports have re-evaluated the diagnostic utility of the glucagon stimulation test (GST) which elicits an ACTH-dependent cortisol response. Glucagon works by stimulating the release of GH and ACTH through a hypothalamic mechanism.^5^

Little information is available in the literature regarding a prevalence of CAI in TM. Poomthavorn et al.^6^ reported a prevalence of 80.7% in TM patients using ITT. Huang et al.^7^ reported a prevalence of 60 % using GST. The prevalence was higher in males vs. female patients (92% vs. 29%, p=0.049). Ten of 11 subjects who failed the GST subsequently demonstrated normal ACTH and cortisol responses to ovine corticotropin-releasing hormone (oCRH) stimulation test, indicating a possible hypothalamic origin to their AI.^7^. Deficient patients had lower liver iron content (LIC) and smaller pituitary volume (p = 0.08 and 0.11, respectively) than those with normal cortisol response.^7^

In both studies, a peak total cortisol = or > 20 μg/dl after ITT, and = or > 18 μg/dL after GST, were considered as normal.^6^,^7^ Nevertheless, the optimal cutoff for the diagnosis of CAI after GST is still a matter of debate. Recently, the sensitivity and specificity of GST have been studied in 49 adult patients after trans-sphenoidal surgery. ROC analysis revealed an upper cut-off of 21.7 μg/dL (599 nmol/l) with 100% sensitivity and 32% specificity for AI while the lower cut-off (10 μg/dL; 277 nmol/l) had a specificity > 95% and a sensitivity of 72%.^8^ Similar results were also reported by Hamrahian et al.^9^ The Authors, using GST for diagnosing AI in adults, established a lower cortisol cut-off point (9 μg/dL= 42.7 nmol/L; 92 % sensitivity and 100 % specificity) after GST.^9^ A cortisol response between 9 and 18 μg/dL (250 nmol/l and 500 nmol/l) to glucagon stimulation was considered a “gray zone).^9^

We undertook the present study to evaluate the adrenal response to GST test in 17 adult patients (8 males) with TM. Their ages ranged from 25 to 53 years (mean 35.2±7.4 yr). All patients were on regular blood transfusions and iron chelation therapy with deferoxamine, deferiprone or deferasirox. Three males and two females were on hormone replacement therapy with sex steroids for hypogonadotropic hypogonadism or secondary amenorrhea (1 patient). Three patients (2 females) received daily levothyroxine for primary hypothyroidism. Thirteen patients were carriers of HCV virus, and two were on treatment for cardiac disease. Serum ferritin was measured by the electrochemiluminescence immunoassay.

Sampling was conducted during the patients’ routine clinical care for endocrinal evaluation. The GST was performed by intramuscular injection of 1 mg of glucagon. Blood samples were drawn every 30 minutes from baseline to 180 minutes for glucose, cortisol and growth hormone (GH) determinations.

Using a cut-off level of 9 μg/dL (250 nmol/l ) as evidence of CAI^14^,^15^ one female patient (5.8%), 26 years old with a serum ferritin of 1288 ng/ml had a poor cortisol response (7.9 μg/dL = 217.9 nmol/l ). Six patients (35.2%; 33± 4.1 yr; serum ferritin 895 ± 434 ng/ml; range: 360–1594 ng/ml) had a normal cortisol response. The remaining 10 patients (58.8%; 37.6± 8.3 yr; serum ferritin 1157 ± 1158 ng/ml; range: 217–3064 ng/ml) a cortisol response (14.4 ± 2 μg/dL = 397.2±55.1 nmol/l) in the “gray zone” (between 9 and 18 μg/dL=250 nmol/l and 500 nmol/l) 9 and a cortisol rise of less than 6.1 μg/dL (170 nmol/L) respect to the basal level. Using a cut-off level of 18 μg/dL (500 nmol/l) 64.7% of our TM patients had an adrenal insufficiency (5/11- 45.4% were males).

The maximum cortisol release, during GST, was observed after 30 minutes in 5 patients, after 60 minutes in 4 patients and after 120–180 min in the remaining 8 patients. No differences in cortisol response were observed between males and females. The side effects reported in the majority of patients were mild flushing, nausea, and headache. One male patient had hypotension.

Five TM patients with a peak cortisol level between 9 and 18 μg/dL (250 nmol/l and 500 nmol/l) after GST, consented to receive an ITT. The test was done giving 0.1 IU/kg of regular insulin (Actrapid, Novo Nordisk) intravenously to achieve blood glucose below 50% of fasting level or less than 40 mg/dl. Blood samples for cortisol and glucose were collected at 0,15, 30, 45, 60, 90, and 120 min. The maximum interval between the two dynamic tests was 2 months. All procedures were carried out between 0800 and 0830 h after overnight fasting. Serum cortisol levels were measured with soli-phase competitive chemiluminescent immunoassay; the inter- and intra-assay CVs were below 6.7%.

Using ITT, 2 out of the 5 patients (1 male ad 1 female, aged 45 and 37 yr with a serum ferritin of 644 ng/ml and 1221 ng/ml, respectively) had low peak cortisol response (16.1 μg/dL=444 nmol/l = and 14.4 μg/dL=397.2 nmol/l, respectively) confirming the central origin of AI. Both patients were asymptomatic and had a basal cortisol level between 9 and 10 μg/dL (248–275 nmol/l) before GST and/or ITT. The adrenocorticotropin hormone (ACTH) level was not measured in both patients.

Nine patients (52.9%); 2 with normal cortisol response and 7 with cortisol response in the “gray zone” had a GH peak after GST< 3.0 μg/L, a value compatible with severe growth hormone deficiency (GHD).

Deposition of iron in the pituitary gland leads to hypogonadotropic hypogonadism and other manifestations of hypopituitarism, including central hypothyroidism and growth hormone (GH) deficiency.^10^–^13^ Therefore, it might also reduce ACTH secretion producing secondary CAI. Although in our study no correlation was observed between basal cortisol level and serum ferritin (r: 0.498;p: NS).The prevalence of AI appears to be more in patients with greater transfusion burden, poor linear growth and wasting. 1,2,14

In conclusion, the identification of TM patients with subtle abnormalities of the HPA is mandatory to avoid a potential adrenal crisis during stressful conditions. Although GST represents an alternative to the ITT as a screening test for CAI because of its accessibility, lack of influence by gender and relatively few contraindications, further larger studies are required to accurately assess the cut-off cortisol level for diagnosing an AI. Fifty-eight percent of our TM patients had a cortisol response in the “gray zone”, after GST (between 9 and 18 μg/dL (250 nmol/l and 500 nmol/l). Two out of the 5 patients with a gray zone response mounted a subnormal response after ITT (CAI).

We believe that the test of choice for diagnosing CAI requires knowledge of the available reference assays and the vagaries of each test. A flow chart for screening and diagnosing adrenal insufficiency in thalassemia is given in figure 1. Furthermore, GST should be cautiously used in TM patients with co-morbidities, including vascular and cardiac diseases, which increase the potential risk of GST.

Yours faithfully

## Figures and Tables

**Figure 1:**
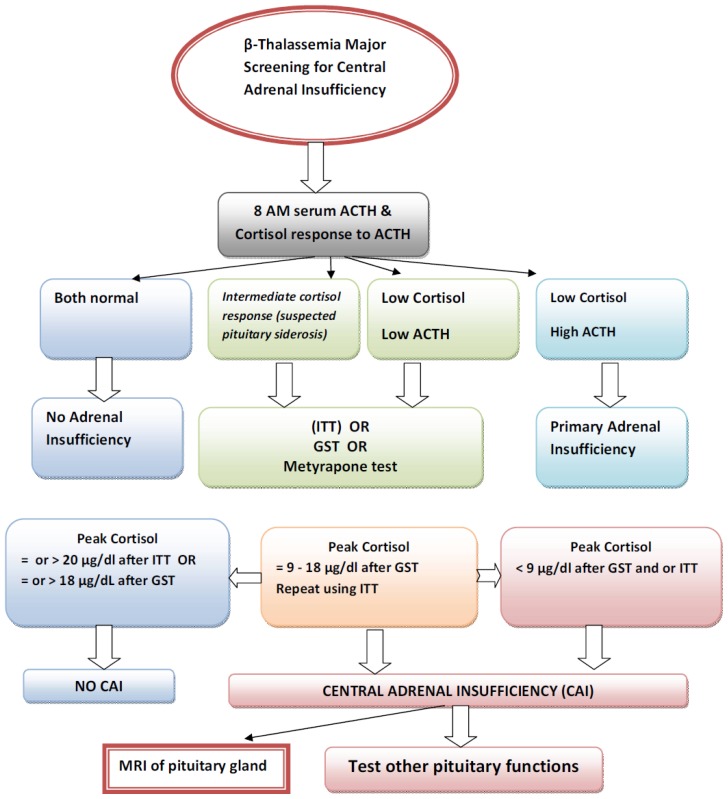
Flow chart for screening and diagnosing adrenal insufficiency in thalassemia
